# Synthetic Strategies in the Preparation of Phenanthridinones

**DOI:** 10.3390/molecules26185560

**Published:** 2021-09-13

**Authors:** Rajeshwar Reddy Aleti, Alexey A. Festa, Leonid G. Voskressensky, Erik V. Van der Eycken

**Affiliations:** 1 Organic Chemistry Department, Science Faculty, RUDN University, Miklukho-Maklaya St., 6, 117198 Moscow, Russia; rajeshwar.ukzn@gmail.com (R.R.A.); festa_aa@pfur.ru (A.A.F.); lvoskressensky@sci.pfu.edu.ru (L.G.V.); 2 Laboratory for Organic & Microwave-Assisted Chemistry (LOMAC), Department of Chemistry, University of Leuven (KU Leuven), Celestijnenlaan 200F, B-3001 Leuven, Belgium

**Keywords:** *N*-methoxy benzamides, benzanilides, 2-phenyl benzamides, ketoximes and aldoximes, C–H bond activation, C–C and C–N bond activation, arynes, carbonylation, oxidation

## Abstract

Phenanthridinones are important heterocyclic frameworks present in a variety of complex natural products, pharmaceuticals and displaying wide range of pharmacological actions. Its structural importance has evoked a great deal of interest in the domains of organic synthesis and medicinal chemistry to develop new synthetic methodologies, as well as novel compounds of pharmaceutical interest. This review focuses on the synthesis of phenanthridinone scaffolds by employing aryl-aryl, *N*-aryl, and biaryl coupling reactions, decarboxylative amidations, and photocatalyzed reactions.

## 1. Introduction

The phenanthridinone skeleton is a common structural moiety found in bioactive alkaloids from many sources, such as oxynitidine [1], crinasiadine [2], narciclasine [3], narciprimine [4], phenaglydon [5], pancratistatin [6], oxoassoanine [7], pratosine [8], anhydrolycorinone [9], and lycoricidine [10]. Owing to the many structural derivatives of phenanthridines [11], their synthesis has been known since the beginning of the 20th century [12]. There are many classical methods available, e.g., via the Schmidt reaction [13,14,15,16], the Ullmann reaction [17], and the Beckmann rearrangement reaction [18,19], dienone-phenol rearrangement [20], and intramolecular rearrangement reaction [21]. In addition, phenanthridinones can be constructed through dichromate oxidation [22], internal cyclisation of a benzyne intermediate [23,24,25], and other conventional methods [26,27]. However, these classical reactions have their limitations due to the requirement of additional steps for the synthesis of the key starting materials, and the generally poor to moderate overall yields. Promptly, these techniques were replaced by using non-traditional methods employing environmentally friendly chemicals, microwave-assisted reactions and catalytic approaches. These more recent techniques offer numerous advantages like shorter reaction times, improved yields, acceleration of sluggish transformations, and cleaner reaction products.

**Scheme 1:**
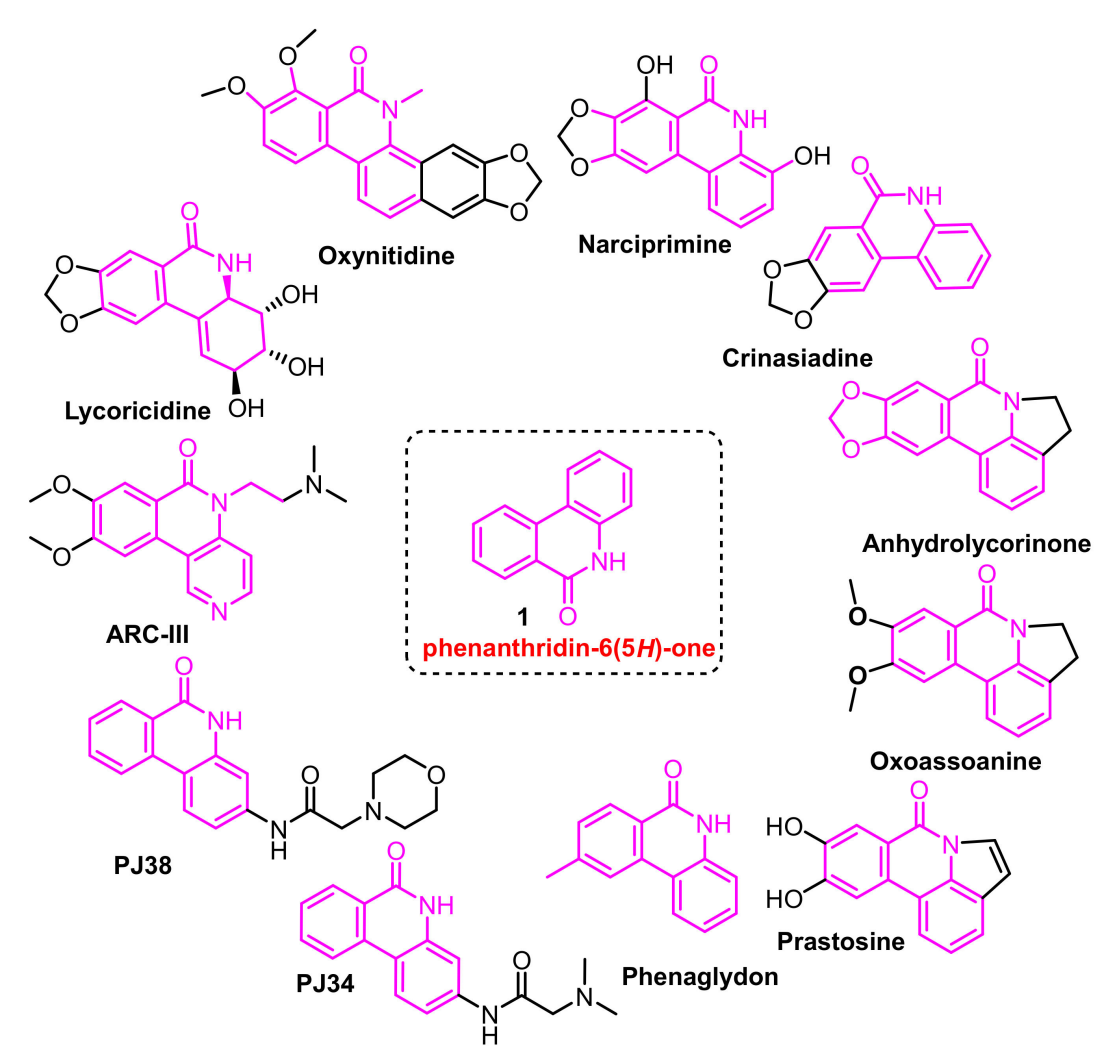
Selected examples of biologically important phenanthridinones.

Phenanthridinones are important heterocycles, as they are used as prospective agents for anticancer treatment [28,29,30], and for antiviral and nerve disorders [31,32]. They possess different pharmacological properties of current interest as a class of HMG-CoA reductase inhibitors [33], anti-HIV [34], anti-hepatitis C virus [35], immunomodulatory activity [36], antibacterial and antifungal activities [37], and treatment of ischemic injuries [38] and other activities [39,40]. Interestingly, they are also estrogen receptor modulators (SERMs) [41], tyrosine protein kinase inhibitors [42], and neurotrophin activity enhancers [43]. Phenanthridinones are known to be inhibitors of poly ADP-ribose polymerase (PARP) family proteins [44,45,46,47,48,49,50,51]. PJ34 [N-(6-oxo-5,6-dihydrophenanthridin-2-yl)-N,N-dimethylacetamide.HCl] is a phenanthridone drug which is a selective inhibitor of poly(ADP-ribose) polymerase [52,53,54]. PJ38 is yet another lead molecule of the phenanthridinone family, which is active as a PARP inhibitor. The synthetic bioactive analogue ARC-111 is well known for its pharmacological activities, and is reported as topoisomerase-1 targeting antitumor agent [55] (Scheme 1).

This review focuses on modern techniques like transition metal-catalyzed coupling reactions, transition metal free coupling reactions, aryne-mediated coupling reactions, amidation reactions through decarboaxylation, photo-induced reactions, oxidation reactions, and anionic cycloisomerization reactions for phenathridinone synthesis.

## 2. Simultaneous Aryl–Aryl and *N*–Aryl Coupling Reactions for Phenanthridinone Synthesis

Transition metal-mediated aryl–aryl and *N*–aryl bond forming reactions are powerful synthetic tools for constructing highly complex molecules [56,57]. They have been recently employed in the key steps of the total synthesis of nitrogen containing natural products, as well as for the construction of heterocycles in medicinal chemistry in a one-pot fashion or multi step processes due to high efficiency and compatibility with numerous functional groups. One-pot C–C/C–N bond forming reactions have been used in drug discovery [58]. In this section, simultaneous aryl–aryl and *N*–aryl coupling reactions are described.

### 2.1. C–C and C–N Bond Formation via a One-Pot Synthesis

**Scheme 2:**
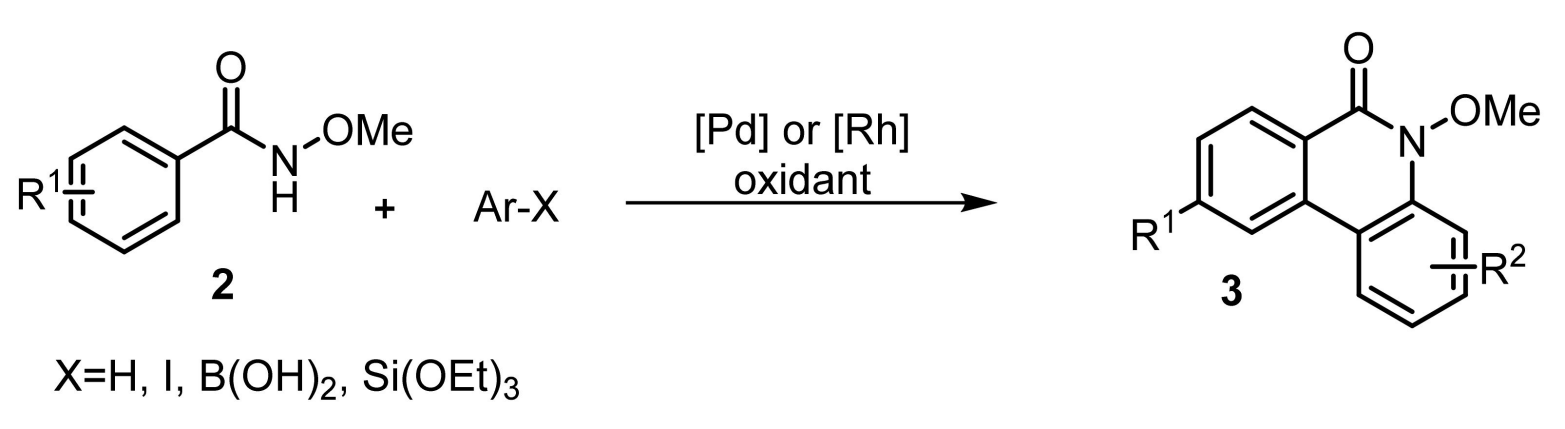
Phenanthridinone preparation from *N*-methoxybenzamides.

Simultaneous C–C and C–N bond forming reactions played pivotal roles for the synthesis of biologically relevant phenanthridinones. The most atom efficient protocols are based on one or more C–H-activation steps, avoiding excessive use of halogenated derivatives. The Snieckus group and Rault group prepared phenanthridinones by using transition metal-catalyzed C–C and C–N bond formation coupling reactions in a two-step process [59,60]. The use of *N*-methoxybenzamides **2** became a fruitful direction for the development of C–H activation methodology (Scheme 2). In 2011, Wang and colleagues demonstrated the synthesis of biologically important phenanthridinones through a one-pot formation of C–C and C–N bonds using palladium-catalyzed dual C–H activation of *N-*methoxybenzamides using phenyliodide and silver oxide as oxidant [61]. Later, the following transformation was realized under the catalysis with binaphthyl stabilized palladium nanoparticles [62]. Further, non-halogenated arenes could be engaged in analogous phenathridinone synthesis [63]. Multiple oxidative C–H bond activation and C–C/C–N bond formation steps in a one pot fashion are the benefits of this process. The utility of the methodology was disclosed through *crinasiadine* natural product synthesis. In continuation, *N*-methoxybenzamides could be annulated with aryl boronic acids [64] or aryl silanes [65] to form phenanthridinones under rhodium-catalysis. In general, the reactions start with the formation of pallada- or rhodacycles, which further react with the coupling partner.

**Scheme 3:**
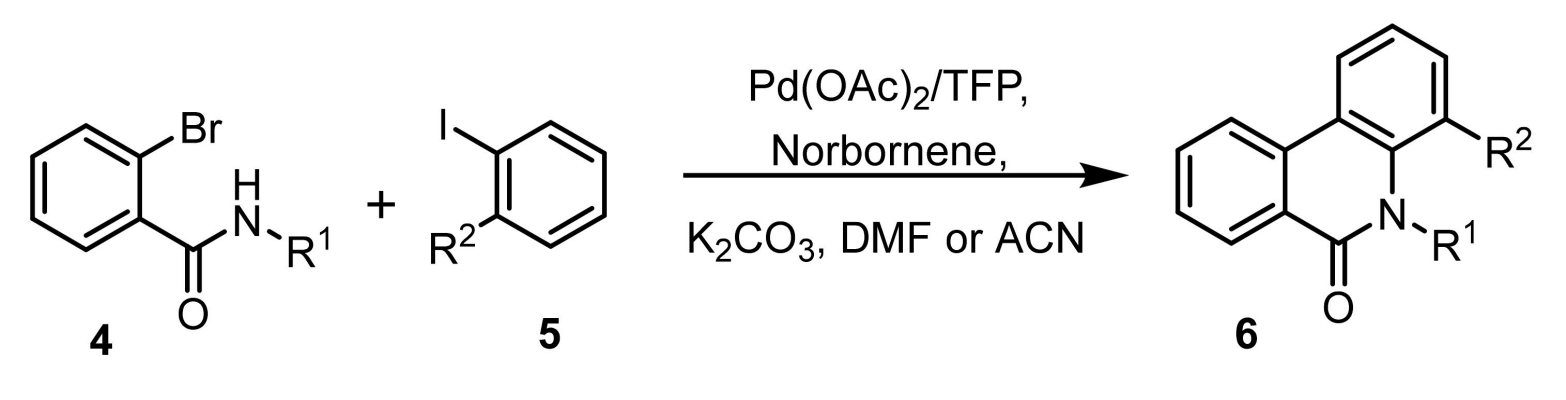
Palladium-catalysed phenanthridinones from 2-bromobenzamides and aryl iodide.

In 2002, Caddick and his colleagues reported the synthesis of phenanthridinones using *o*-halobenzamides through an intramolecular Heck reaction [66]. In 2004, Ferraccioli, Catellani and colleagues reported the synthesis of phenanthridinones **6** using *o*-halobenzamides **4** and aryl iodides **5** as starting materials (Scheme 3) [67]. In this case, the use of norbornene was necessary to mediate the reaction and achieve a C–H activation step. Interestingly, when benzamides without halogen in the ortho position were treated with 1-bromo-2-iodobenzenes under Pd-catalysis, the synthesis of phenanthridinones was successfully achieved in the absence of norbornene [68]. An efficient intermolecular dehydrogenative annulation of aryl iodides and aryl carbamic chlorides was reported for the synthesis of phenanthridinones using Pd-norbornene-catalysis [69].

**Scheme 4:**
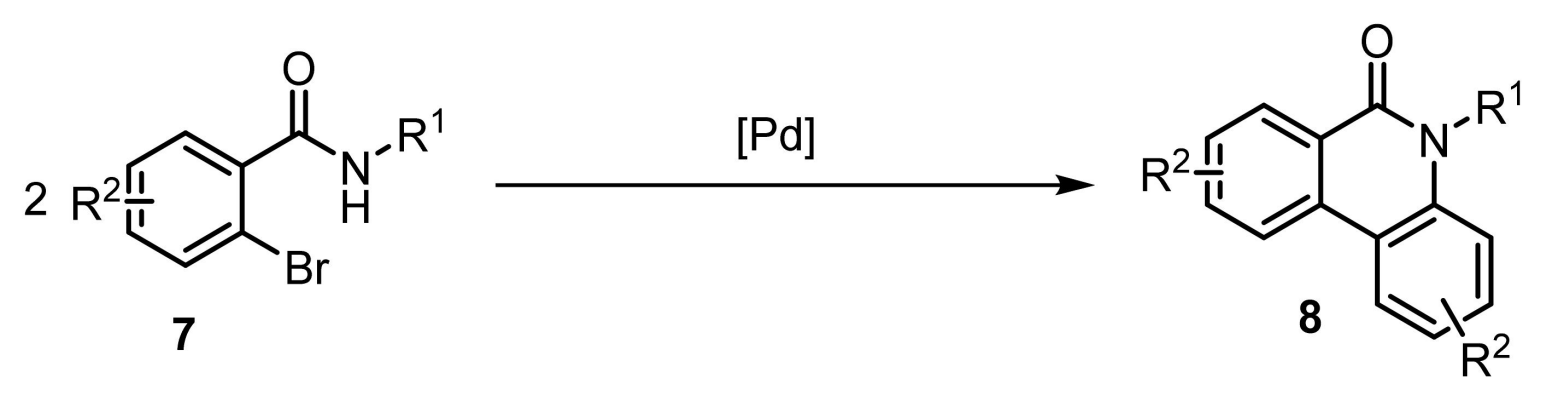
Palladium-catalyzed homocoupling of 2-bromobenzamides.

2-Bromobenzamides **7** could be subjected to Pd-catalyzed homocoupling, delivering phenanthridinone derivatives **8** [70,71,72] (Scheme 4). In 2016, phenanthridinone synthesis was realized starting from *o*-halobenzamides under phosphine-free palladium catalysis in *N*,*N*-dimethylacetamide [73]. Authors also demonstrated this methodology in a scalable process by performing gram scale reactions. Later, Hu and his colleagues attempted the synthesis of *N*–H-phenanthridinones by using palladium-catalyzed intramolecular C–H arylation of 2-halo-2-Boc-*N*-arylbenzamides [74]. A variety of benzamides bearing electron-donating and electron-withdrawing groups can be coupled and produce the corresponding phenanthridinones in moderate to good yields.

**Scheme 5:**
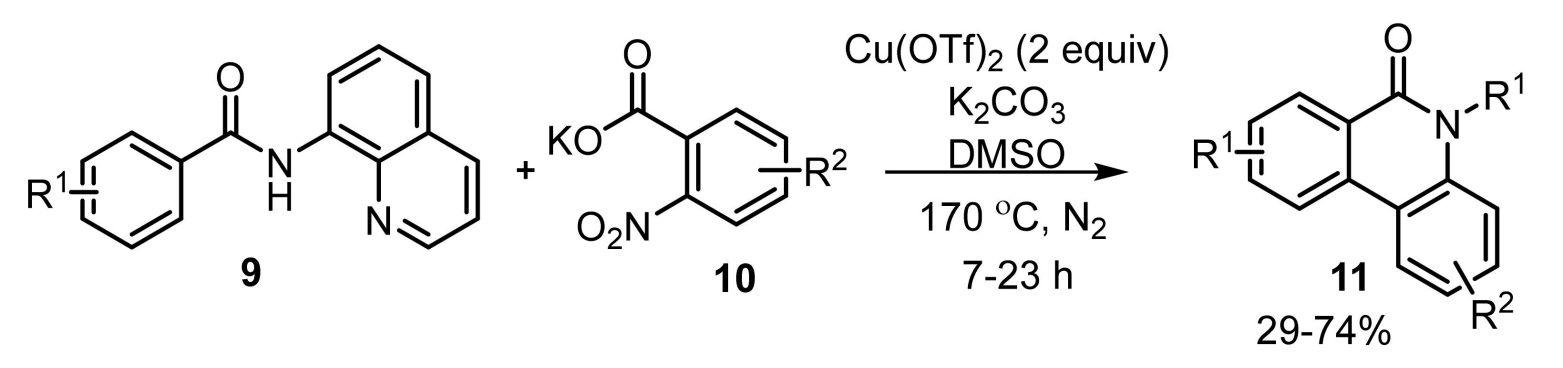
Phenanthridinone preparation using benzamides and *o*-nitrobenzoic acids.

A decarboxylative copper-mediated coupling of benzamides **9** with *ortho*-nitrobenzoic acid salts **10** was realized by Miura and co-workers (Scheme 5) [75]. The C–H-activation step was achieved with the help of a quinol-8-yl directing group. After the decarboxylative coupling, cyclization occurred through nucleophilic substitution of a nitro-group by an amide moiety. The resulting phenathridinones **11** were isolated with moderate yields. Li et al. reported a Pd-catalyzed decarboxylative *ortho-*arylation of *N*-methoxyarylamides using aryl acylperoxides [76].

**Scheme 6:**
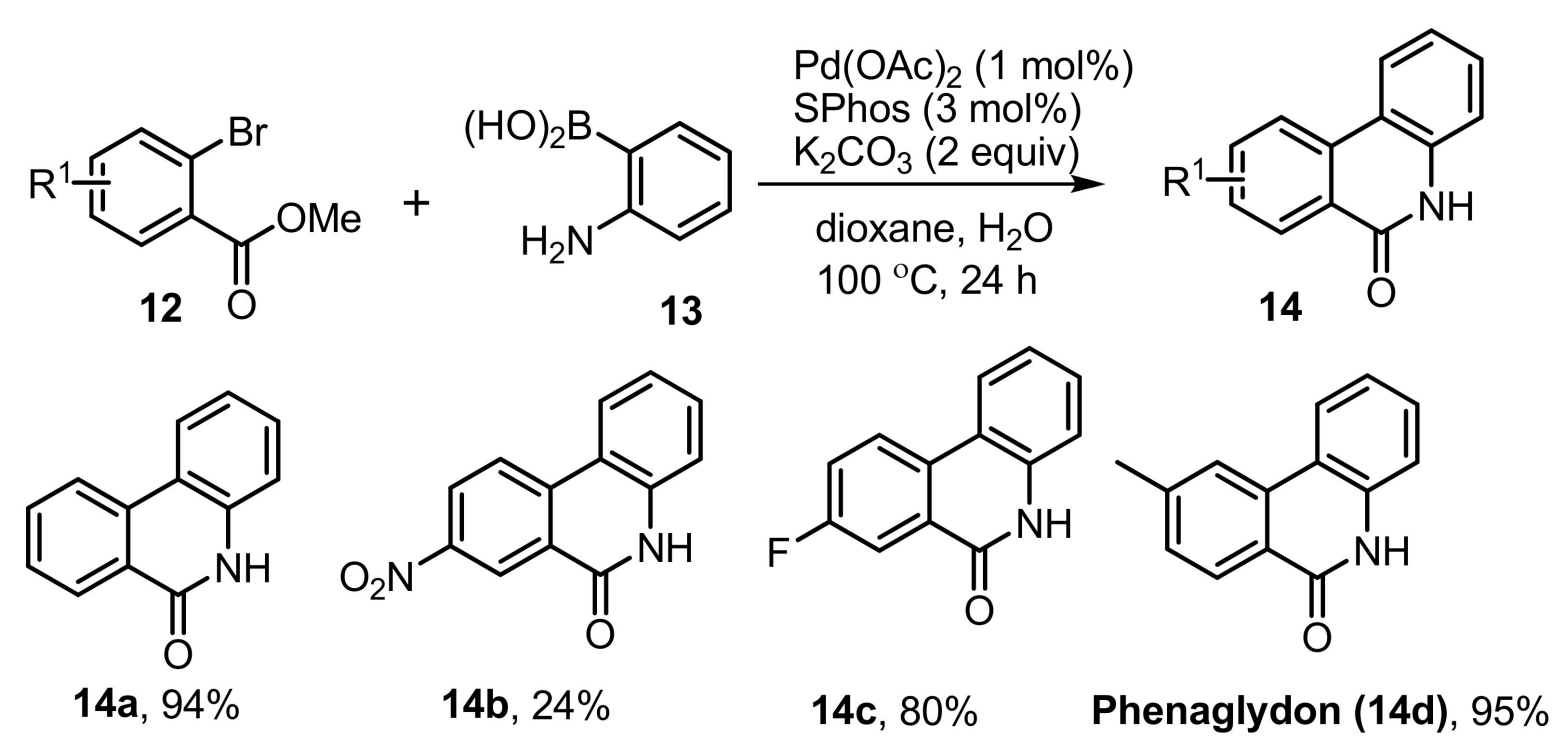
Palladium-catalyzed phenanthridinones from 2-halobenzoate and 2-aminophenylboronic acid.

In 1994, Tour and colleague reported a phenanthridinone synthesis using 2-halobenzoate and aromatic boronic acids using palladium as the catalyst [77]. However, the products were obtained with low yields and the substrate scope was not investigated. Later, Tanimoto et al. established a convenient one-step access to biologically important phenanthridinones **14** using 2-halobenzoate **12** and 2-aminophenylboronic acid **13** in a Suzuki–Miyaura cross-coupling reaction with palladium(II) acetate and 2-dicyclohexylphosphino-2′,6′-dimethoxybiphenyl (SPhos) as precatalysts (Scheme 6) [78]. Interestingly, Kuwata et al. extended this strategy to synthesize the phenanthridinone alkaloids crinasiadine and *N*-alkylcrinasiadines from 2-aminophenylboronic acid and 2-bromobenzoate [79]. Furthermore, 2-halobenzoates were proved to be an efficient substrate for preparing biologically active phenanthridinones [80,81,82].

**Scheme 7:**
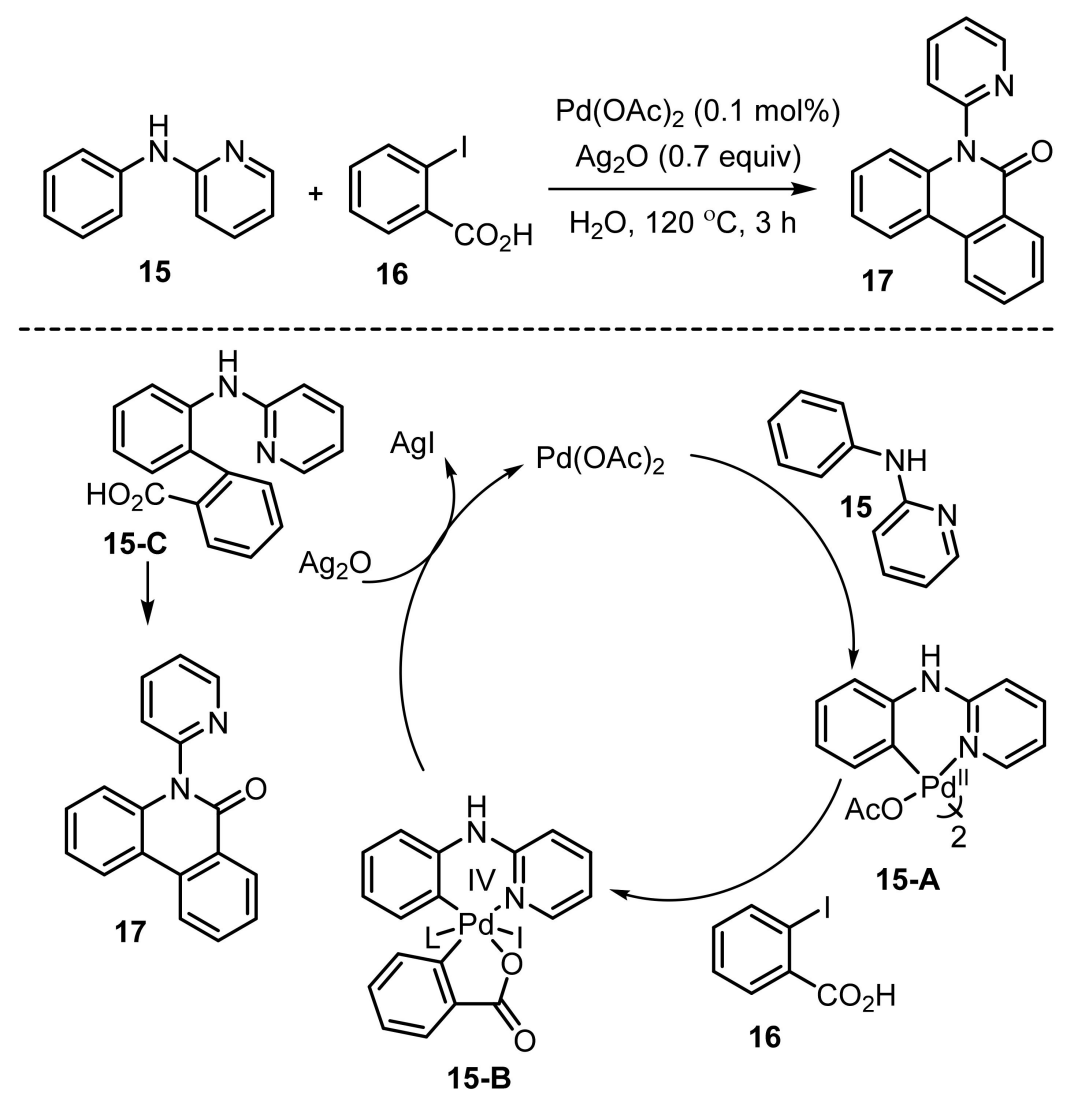
Phenanthridinone preparation from *N*-aryl-2-aminopyridines and 2-iodobenzoic acids.

Recently, Ding et al. reported the synthesis of phenanthridinones **17** through Pd-catalyzed cyclization of *N*-aryl-2-aminopyridine **15** with 2-iodobenzoic acid **16** via C(sp^2^)–H bond activation (Scheme 7) [83]. The advantage of the methodology was in low catalyst-loading, and that the reaction smoothly proceeded in water. The broad substrate scope was demonstrated with high yields comparatively with previous methods. A tentative mechanism, based on experimental results, was proposed by the authors. Primarily, the substrate **15** coordinates with Pd(OAc)_2_ via the pyridine nitrogen atom. A six membered palladacycle dimer complex **15-A** is generated through directed C–H bond activation. Further, a carboxylate-directed oxidative addition generates a Pd(IV) species **15-B**. This is followed by reductive elimination, resulting in the ortho-arylated product **15-C**. Subsequently, the latter undergoes intramolecular acylation to generate **17**.

### 2.2. C–C and C–N Bond Formation via Aryne-Mediated Reactions

**Scheme 8:**
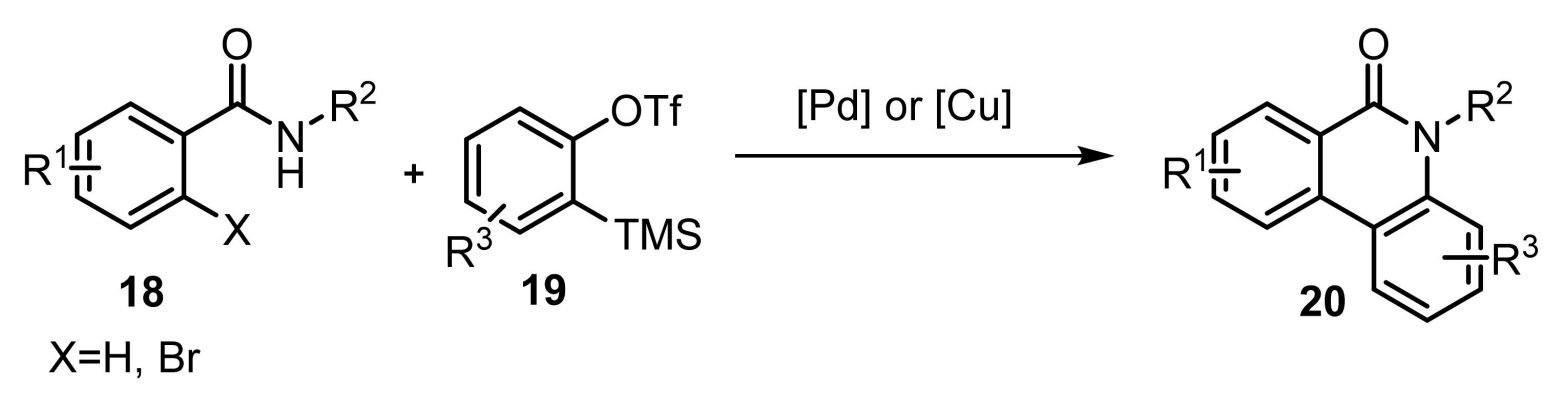
Benzyne-involved preparations of phenathridinones.

**Scheme 9:**
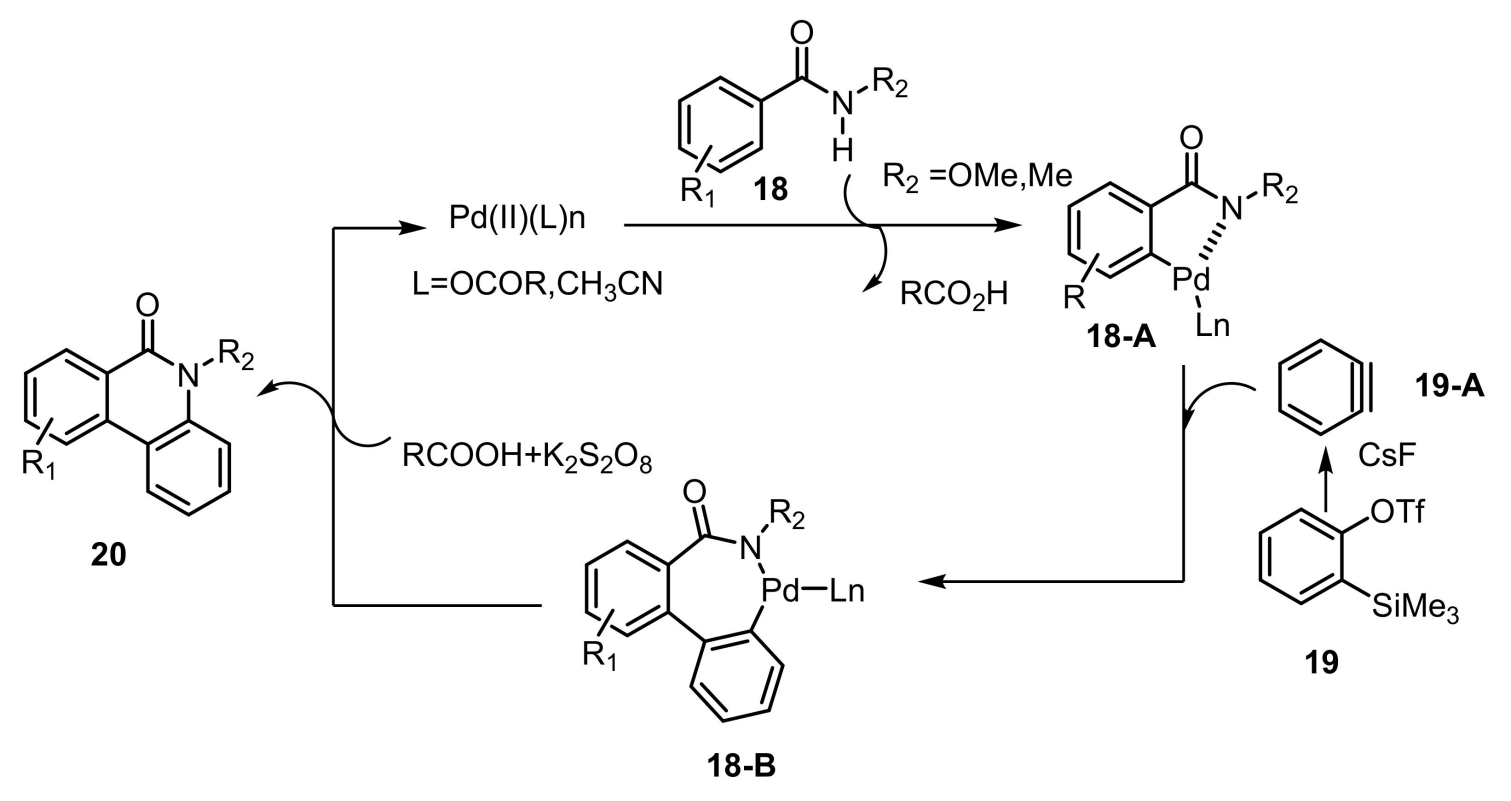
Proposed mechanism for the palladium-catalyzed aryne mediate cyclization.

In 2002, Lu et al. described an aryne-mediated synthetic methodology of substituted *o*-halobenzamides through a palladium-catalyzed annulation for the synthesis of *N*-substituted phenanthridinones in good yields. The advantage of this methodology is the preparation of phenanthridinones in a single step via simultaneous C–C and C–N bond formation, under relatively mild reaction conditions, tolerating a wide variety of functional groups [84]. Later, Jeganmohan and colleagues applied this concept on methyl or methoxy benzamides **18** and reported an aryne mediated cyclization using *o*-(trimethylsilyl)aryl triflate **19** in the presence of Pd(OAc)_2_, organic acid and K_2_S_2_O_8_ in CH_3_CN yielding tricyclic phenanthridinone derivatives **20** in good yields. The scope of the catalytic reaction was examined with a variety of substituted *N*-methoxy benzamides (Scheme 8) [85]. Initially, a five membered palladacycle **18-A** forms by the coordination of *N*-methoxy benzamide **18** to the Pd(0) species followed by *ortho*-metalation. Coordinative insertion of the benzyne intermediate **19-A** into intermediate **18-A** forms a seven membered palladacycle **18-B.** This undergoes reductive elimination, as well as C–N bond formation using RCOOH, K_2_S_2_O_8_ with regeneration of the Pd catalyst (Scheme 9). Furthermore, the utility of benzyne precursors was reported using palladium, copper, and nickel catalysts [86,87,88,89,90].

### 2.3. C–C and C–N Bond Formation via Carbonylative and Carboxylative Reactions

**Scheme 10:**
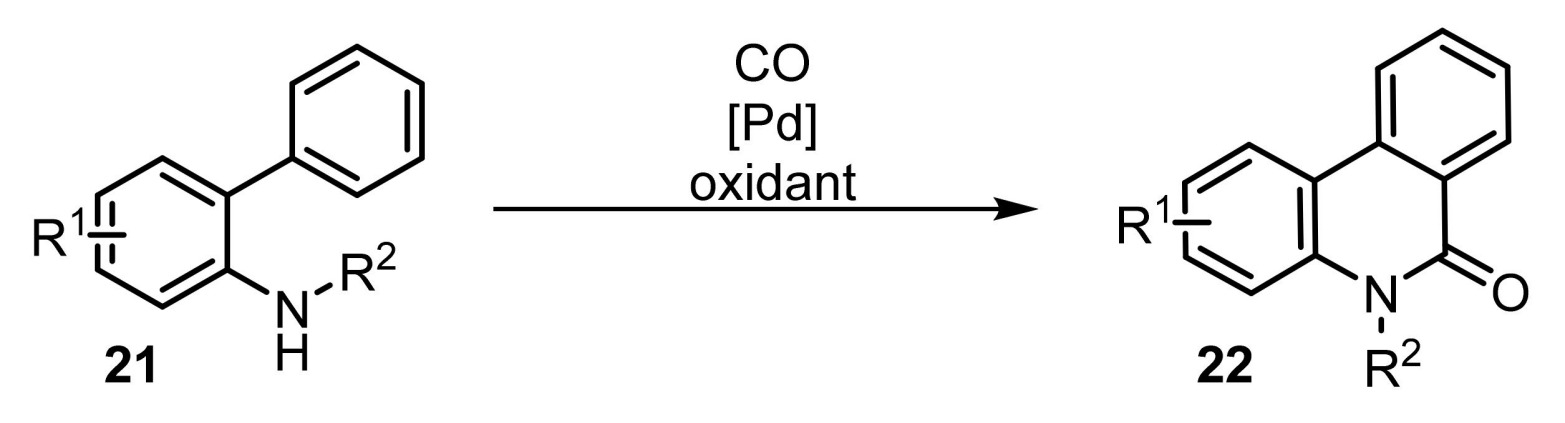
Pd-catalyzed carbonylative synthesis of phenanthridinones.

In 2013, Zhang [91] and Zhu [92] concurrently described a phenanthridinone synthesis through carbonylation of biphenyl-2-amines **21** employing a combination of Pd(II) and Cu(II) salts under a carbon monoxide atmosphere (Scheme 10). Zhang and co-workers succeeded in developing a catalytic system that can avoid the formation of urea as side product, using a combination of Pd(OAc)_2_ and Cu(II) trifluoroacetate in 2,2,2-trifluoroethanol. The substrate scope of the reaction was verified using various electron rich and electron deficient biphenylamines. Electronic rich biphenylamines gave higher yields compared to electronic deficient biphenylamines [91]. In the case of Zhu’s work, 1 equiv of TFA was needed for smooth conversion [92]. Moreover, Chuang and colleagues used only a palladium catalyst for oxidative insertion of carbon monoxide on *N*-sulfonyl-2-aminobiaryls to furnish phenanthridinone **22** [93]. Interestingly, DMF could also be used as a one-carbon-source instead of toxic carbon monoxide [94]. Ling and colleagues reported a CoCl_2_-catalyzed carbonylation of *ortho*-arylanilines in the presence of diazadicarboxylates as a carbonyl source and oxygen as oxidant [95]. 10-Hydroxy-10,9-borazarophenanthrenes could be converted into phenanthridinones, including phenaglydon, through Pd(II)-catalyzed carbonylation [96].

**Scheme 11:**
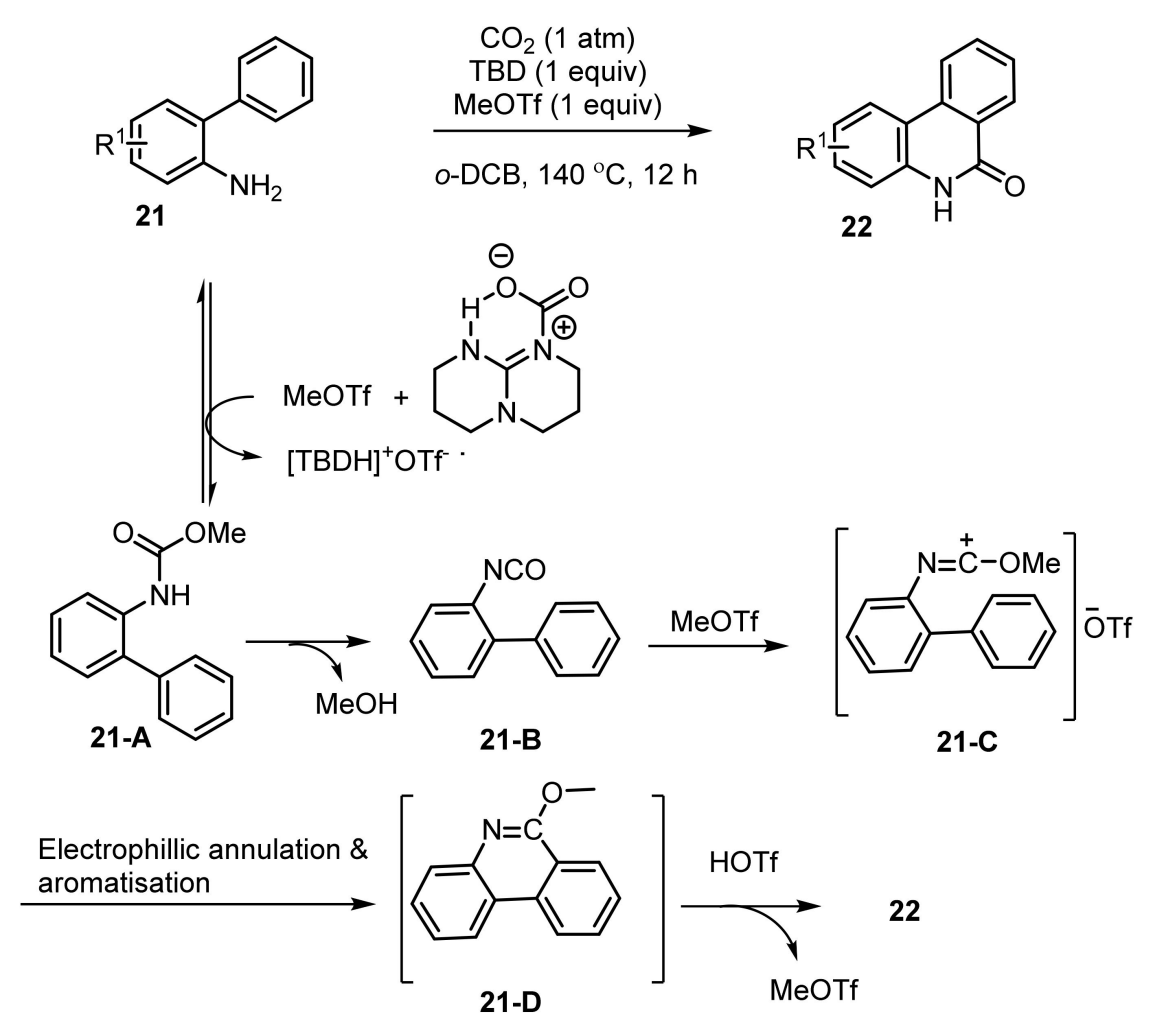
Transition metal free carboxylative cyclization of *o*-arylanilines.

The use of non-toxic CO_2_ as a carbonyl source for phenanthridine formation could be advantageous. For example, o-arylanilines **21** could be transformed into phenanthridinones **22** under a CO_2_ atmosphere using transition metal free conditions (Scheme 11) [97]. Substrates bearing an electron-donating group on the aryl moiety, demonstrated higher reactivity to furnish **22** with higher yields, while electron-withdrawing groups gave lower yields. Based on experimental results, a plausible mechanism was proposed. Initially, the *o*-arylaniline **21** reacted with the adduct of 1,5,7-triazabicyclo[4.4.0] dec-5-ene (TBD) and CO_2_ to produce the carbamate **21-A** with the aid of MeOTf. Release of MeOH generated isocyanate **21-B** at high temperature. Subsequently, internal cyclization of intermediate **21-C** resulted in the formation of **21-D**, which upon hydrolysis gave phenanthridinone **22.** In 2021, aryisocyanates **21-B** were directly transformed into phenathridinones under the action of catalytic amounts of MeOTf in DCE. That approach was used for the synthesis of *amaryllidaceae* alkaloids [98].

**Scheme 12:**
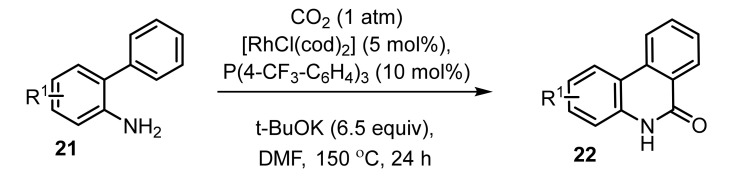
Rh-catalyzed carboxylative cyclization of *o*-arylanilines.

In 2019, Gao et al. developed an amino group-assisted C–H carboxylation of 2-arylanilines with CO_2_ through Rh(I) catalysis under redox neutral condition (Scheme 12) [99]. The reaction was carried out in the presence of a phosphine ligand and *^t^*BuOK as base. The advantage of the methodology is that no external oxidant was needed. The reaction started with the oxidative addition of Rh(I), followed by a reductive elimination of HX to generate a rhodacycle, which underwent carboxylation with CO_2_. The resulting Rh carboxylate cyclized into the target product. Only *N*-unsubstituted 2-phenylanilines **21** participated successfully in the reaction.

## 3. C–C Coupling Reactions for Phenanthridinone Synthesis

Arylation of arenes has emerged as a powerful tool for the synthesis of biaryl compounds, which led to a variety of bioactive molecules [100,101]. Biaryl coupling has shown a prominent strategy to prepare bioactive phenanthridinones. The synthesis of phenthridinones through C–C coupling reactions is discussed in this section.

**Scheme 13:**
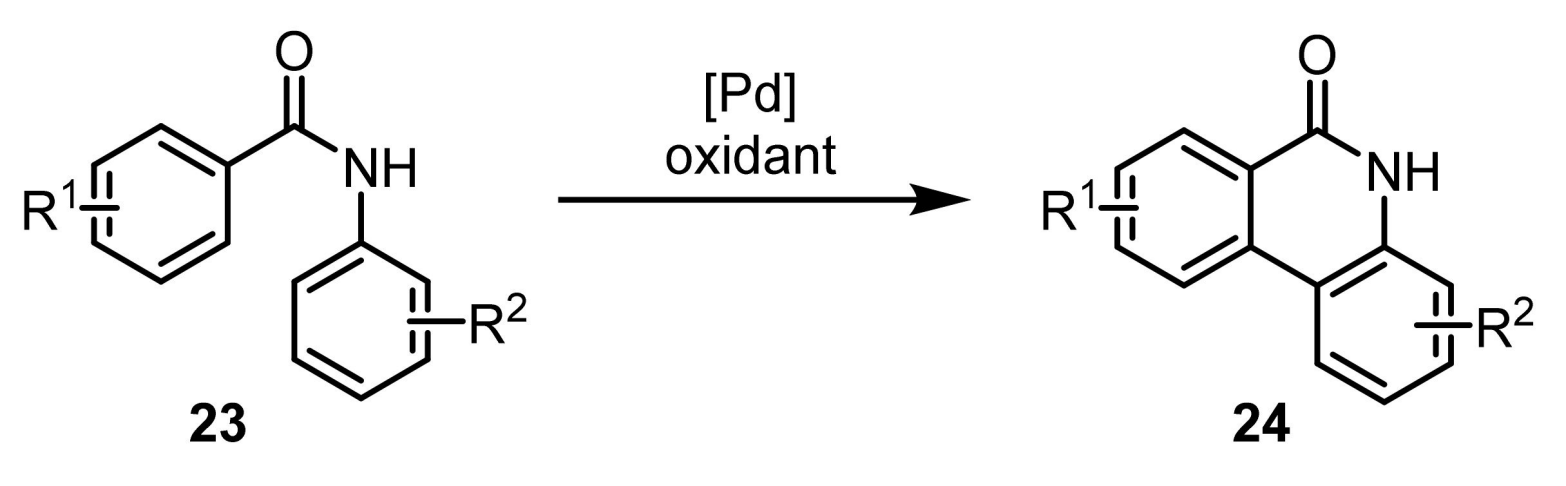
Biaryl dehydrogenative coupling.

Benzanilides with halogen substituent in the *ortho*-position were extensively used for the preparation of phenathridinones [102,103,104,105,106,107,108]. Later, a dehydrogenative approach with halogen-free compounds was developed. Dong and co-workers reported an intramolecular *ortho*-arylation of benzanilides **23** [109]. The reaction was performed in DCE under the action of palladium (II) acetate (10 mol%) and Na_2_S_2_O_8_ as oxidant. The addition of TFA was crucial for the transformation. The methodology allowed the synthesis of *N*-methylcrinasiadine natural product in 30% yield. Further, Murakami’s group used molecular oxygen as terminal oxidant for the palladium-catalyzed arylation of benzanilides **23** towards the synthesis of phenanthridinones **24** via oxidative C–H coupling (Scheme 13) [110]. Interestingly, non-substituted benzanilide afforded the phenanthridinone in 89% yield. Chloro- and bromo-groups remained intact, while cyano-substituents were not tolerated under the reaction conditions. Later, Du, Zhao, and-coworkers developed a Cu(II)-promoted conversion of *N*-benzoylated enaminones into 3-hydroxyphenanthridinones [111]. Interestingly, *N*-formyl-2-arylanilines could be cyclized into phenathridinones under Cu(0)-catalysis in the presence of stoichiometric amounts of selectfluor [112].

**Scheme 14:**
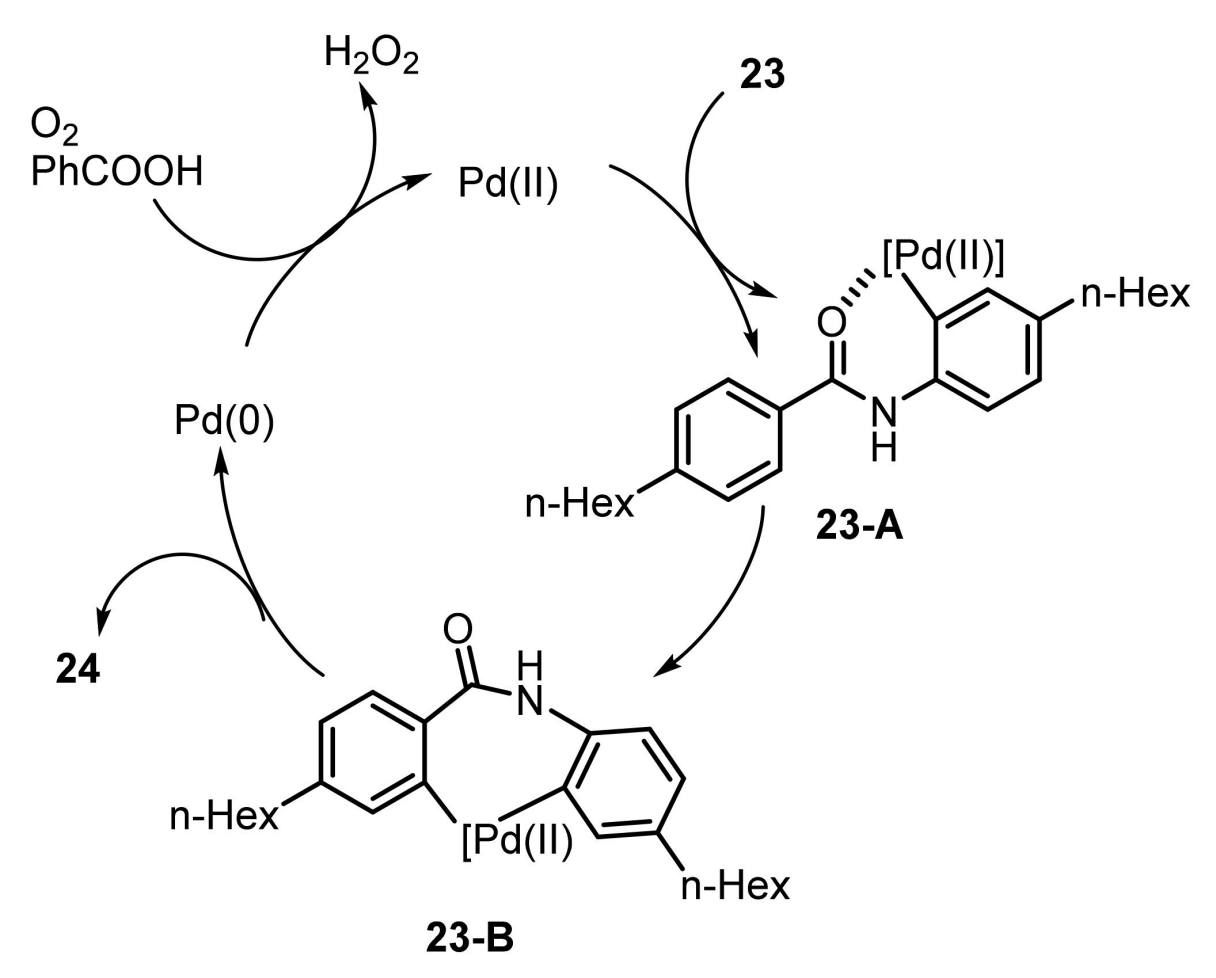
Proposed mechanism for the biaryl dehydrogenative coupling.

The following mechanism was proposed by the authors (Scheme 14) [110]. The palladium (II) species coordinates to the carbonyl oxygen of the amide group of the anilide via *ortho*-palladation, resulting in the formation of the aryl palladium species **23-A**. Un-coordination of the amide bond in intermediate **23-A** was followed by the rotation of the amide bond and placing the *ortho*-position of the benzoyl moiety in close proximity to the palladium center, leading to the second aromatic palladation to generate intermediate **23-B**. Reductive elimination gives phenanthridinone **24** and palladium (0). Palladium (II) was regenerated by the action of molecular oxygen and benzoic acid.

**Scheme 15:**
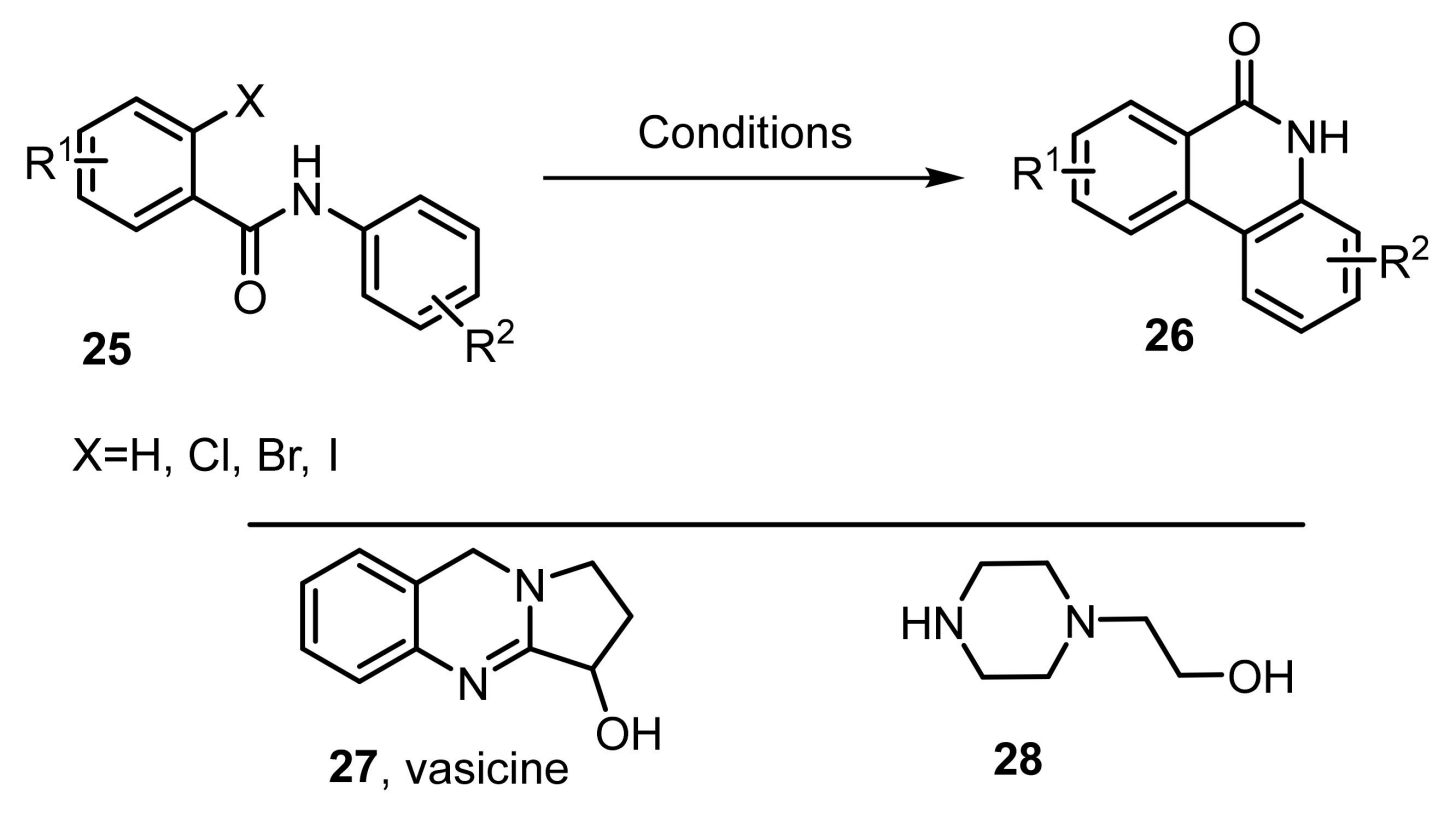
Transition metal free aryl arene coupling of halo benzamides for phenanthridinone synthesis.

Benzanilides **25** could be cyclized into phenathridinones **26** under transition metal free conditions. For instance, benzanilide **25** (X=H) underwent the desired transformation in the presence of phenyliodine (III)-bis (trifluoroacetate) (PIFA) (Scheme 15) [113]. The reaction was possible for electron-rich substrates. Bhakuni et al. developed a transition metal free aryl arene coupling of halo benzamides **25** using KO*^t^*Bu, 1,10-phenathroline as ligand and AIBN as a radical initiator [114]. In 2016, Sharma et al. developed a transition metal-free microwave-assisted methodology utilizing vasicine **27** (a natural product) as a catalyst for the synthesis of phenanthridinones. The reaction proceeded through intramolecular C–H arylation with aryl halides in the presence of KO*^t^*Bu base, under microwave irradiation in sulfolane as the solvent. Remarkably, the reaction worked smoothly with less reactive aryl chlorides and reached completion after 15 min [115]. Bromides could be analogously cyclized in the presence of 1-(2-hydroxyethyl)-piperazine (**28**) and DMAP in mesitylene (Scheme 15) [116].

**Scheme 16:**
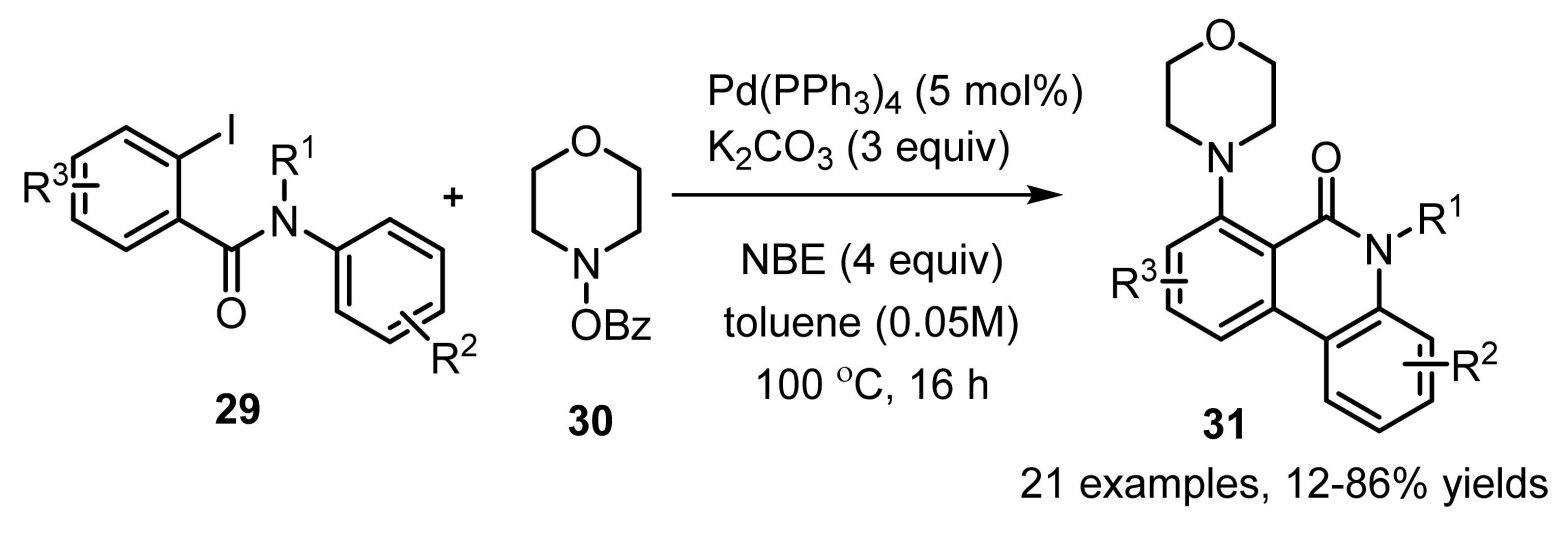
Rh-catalyzed carboxylative cyclization of *o*-arylanilines.

Lautens and colleagues reported a synthesis of remarkable *ortho*-aminated phenanthridinones **31** to be produced by reacting wide scope of benzamides **29** with *O*-benzoyl-hydroxylamine **30** under palladium-norbornene catalysis. Various hydroxylamines could also be used, although less efficiently (Scheme 16) [117].

## 4. *N*–Aryl Coupling Reactions for Phenanthridinone Synthesis

An *N*–Aryl coupling reaction opened avenues for the synthesis of heterocycles, that are abundant in natural products [118]. In addition, *N*–aryl coupling reactions proved to be a good strategy to prepare pharmaceutically important phenanthridinone derivatives. In this section, phenanthridinone synthesis, using metal catalyzed *N*–aryl coupling reactions and transition metal free *N*–aryl coupling reactions, is discussed.

**Scheme 17:**
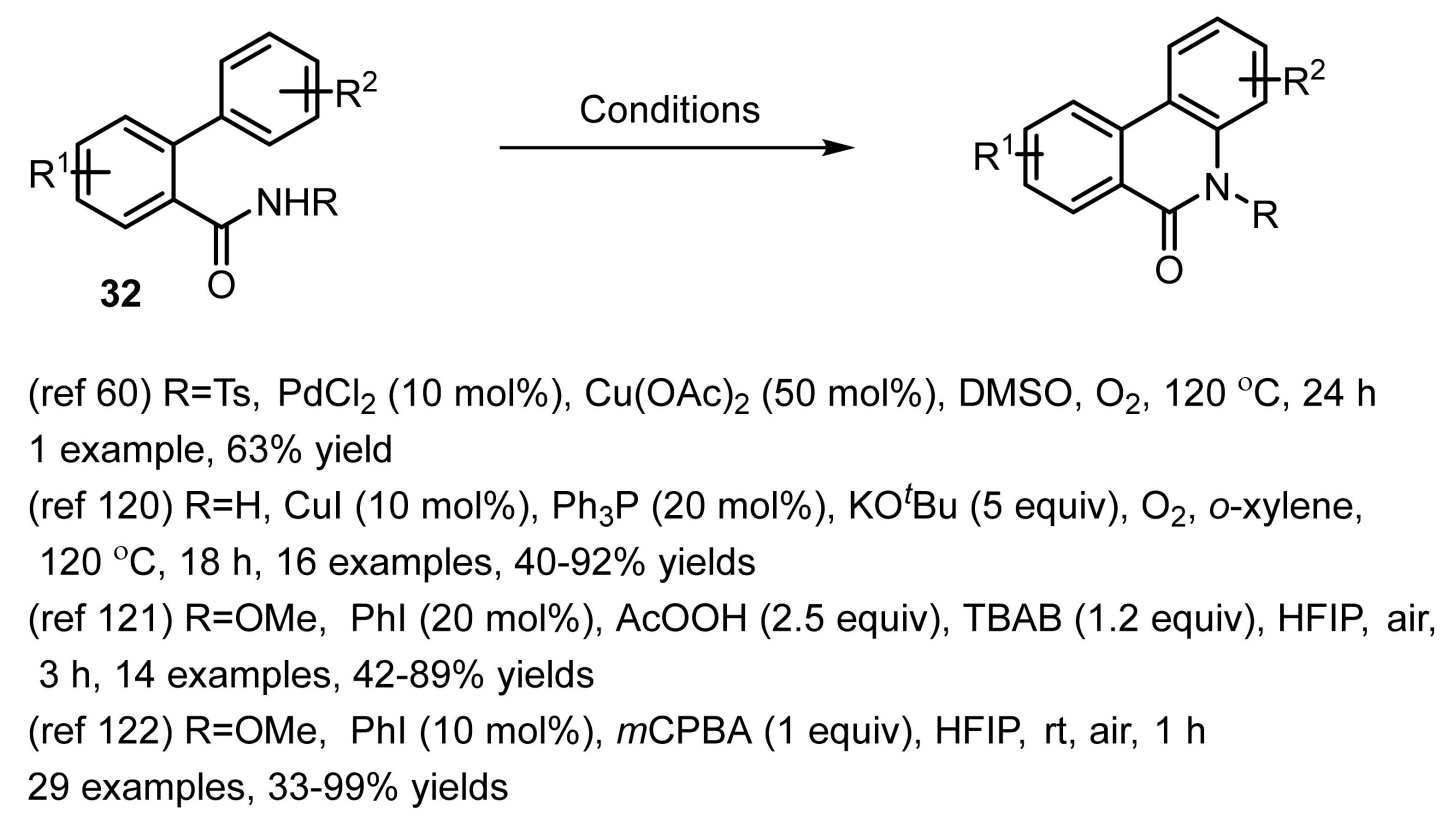
C–H amidations for the synthesis of phenathridinones.

Oxidative intramolecular palladium-catalyzed C–H amination of arenes for the synthesis of phenanthridin-6-one in 63% yield was reported in 2012 [60], employing the procedure for the synthesis of quinolones (Scheme 17) [119]. The removal of the tosyl group took place at the same time. Later, Gui and colleagues developed a copper-catalyzed approach for the preparation of phenanthridin-6-ones from 2-phenylbenzamides **32** (R=H) through an intramolecular *N*-aryl bond-forming process under basic conditions [120]. Ortho-arylation of benzamides proceeded smoothly in the presence of CuI (10 mol%), KO*^t^*Bu (2 equiv), and a phosphine ligand (20 mol%). Heating the reactant in *o*-xylene at 120 °C for 18 h was needed to produce the target molecules in 40%–92% yield. Only *N*-unsubstituted amides could be used. Later, hypervalent iodine-mediated processes for the cyclization of *N*-methoxybenzamides **32** (R-OMe) were developed. Jiang, Xue, and-coworkers used catalytic amounts of iodobenzene and peroxy acids as oxidants [121,122]. The reactions were performed in 1,1,1,3,3,3-hexafluoroisopropanol (HFIP) under an air atmosphere. These consecutive C–H functionalization reactions can be used efficiently to construct 2-substituted-phenanthridinones at room temperature with moderate to high yields. Interestingly, when the reaction was carried out in the presence of TBAB, a bromide was introduced in *para*-position of nitrogen [121]. In addition, it has been recently shown that *N*-methoxy amides could be also cyclized into phenathridinones under electrochemical conditions [123].

**Scheme 18:**
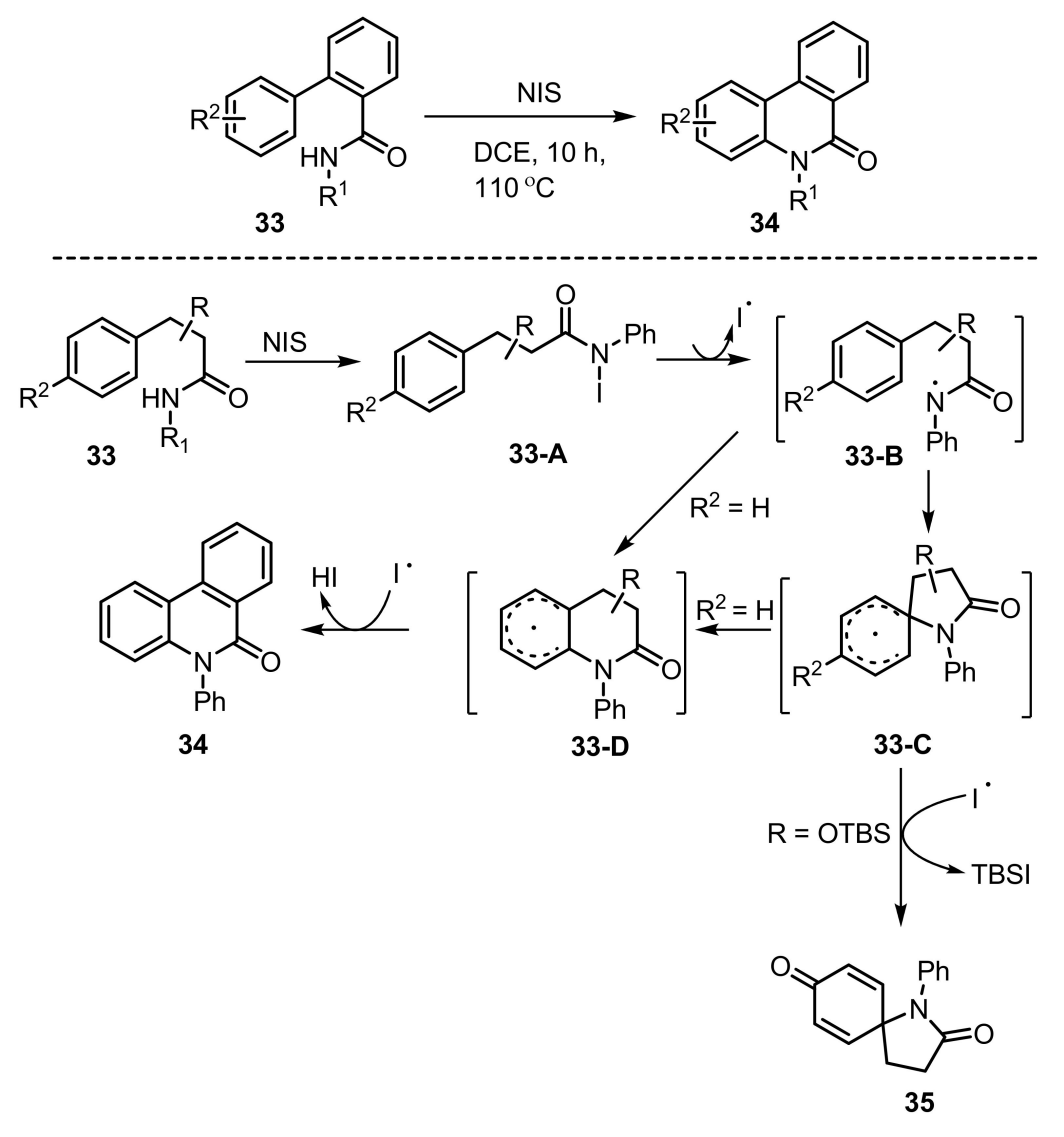
NIS mediated oxidative arene C(sp^2^)–H amidation.

Wang et al. reported an oxidative arene C(sp^2^)–H amidation for the synthesis of phenanthridinones using amide **33** and NIS as iodinating reagent (Scheme 18) [124]. Primarily, *N*-iodosuccinimide reacts with amide **33** to form *N*-iodinated compound **33-A**. It undergoes an *N*–I homolytic cleavage providing amide *N-*radical **33-B** under thermal conditions. Subsequently, 5-exo-cyclization on to the aromatic ring of intermediate **33-B** afforded intermediate **33-C**, or 6-endo cyclization gave benzolactam intermediate **33-D**. Benzolactams could also be obtained through a ring expansion via C–N bond migration. Further rearomatization gave product **34**. When the substrate was bearing OTBS-group, the intermediate **29-C** delivered spirolactam **35.** Later, Verma et al. reported a cyclization of 2-phenylbenzamides for the preparation of phenanthridinones using iodine, succinimide, and di-*tert*-butylperoxide (DTBP) as oxidant [125]. It has been shown that 2′-iodo-[1,1′-biphenyl]-2-carboxamide could be cyclized into phenathridinone under Ni-catalysis in the presence of boronic acid [126].

**Scheme 19:**
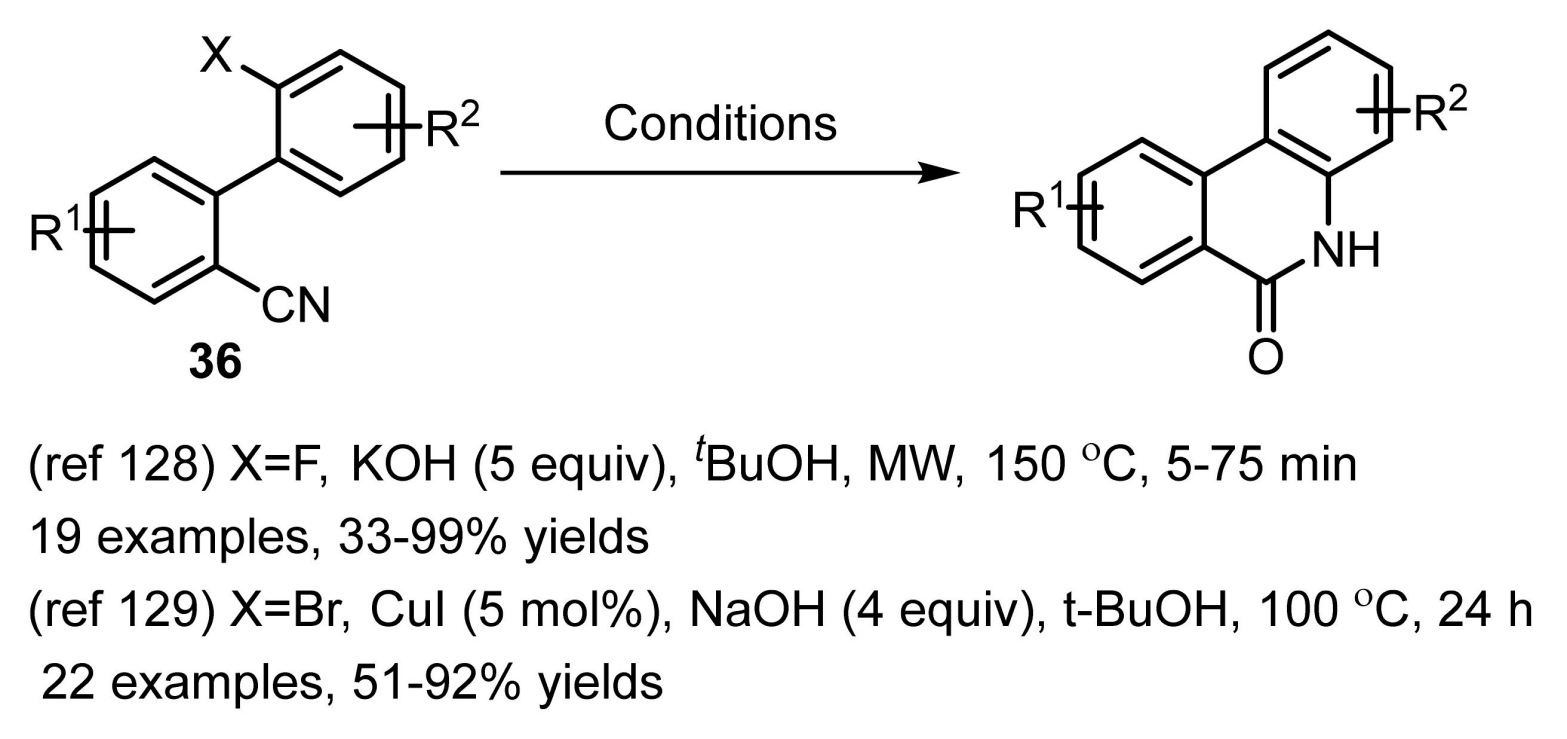
Transformations of 2-arylbenzonitriles.

Other useful substrates for the synthesis of phenathridinones via a C–N bond forming route are 2-arylbenzonitriles **36**. Fabis and-coworkers reported a potassium hydroxide mediated anionic ring closure of fluorine-substituted benzonitriles **36** (X = F) [127,128]. Later, Chen et al. reported a copper-catalysis of annulation of bromide-substituted benzonitriles **36** (X = Br) (Scheme 19) [129]. Furthermore, the utility of copper catalysis was extended to the synthesis of phenathridinone containing bioactive natural products [130,131]. Benzonitriles with an alkyne moiety in *ortho*-position could undergo an anionic cycloaromatization when treated with sodium methylate in methanol, delivering condensed phenathridinones [132,133].

## 5. Decarboxylative Reactions for Phenanthridinone Synthesis

**Scheme 20:**
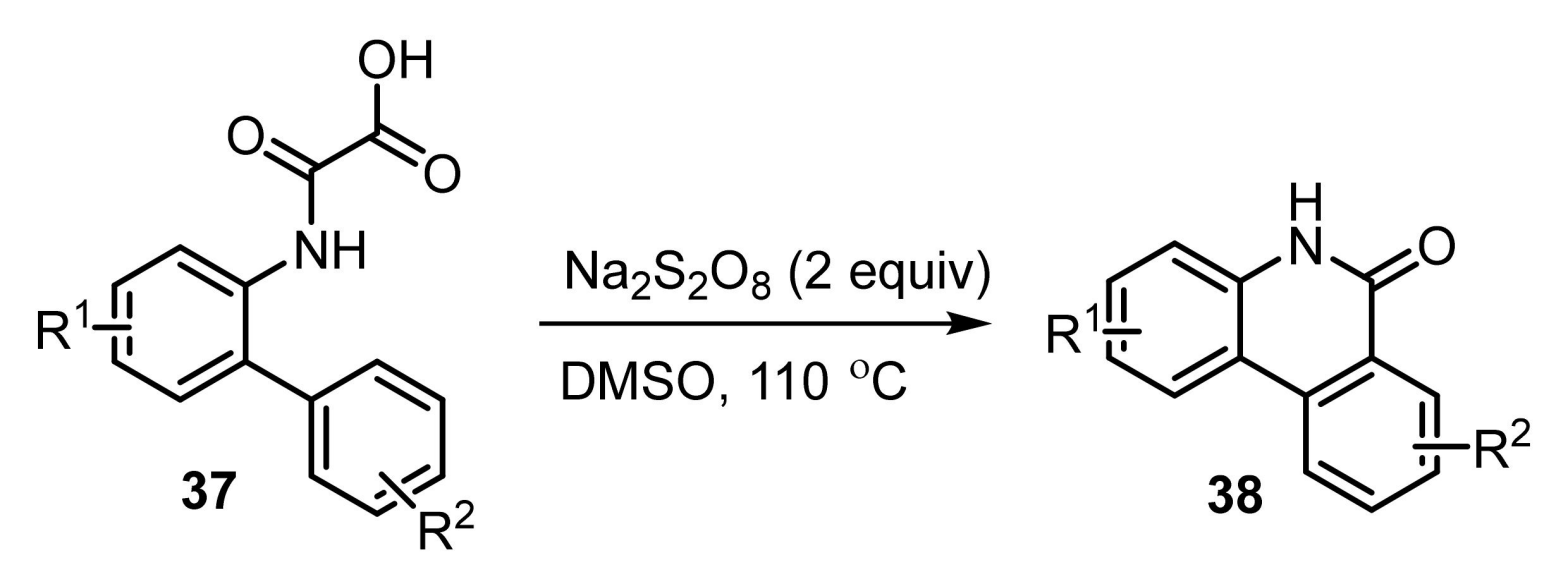
Transition metal free synthesis of phenanthridinones from biaryl-2-oxamic acids.

Efficient C–C bond formation via transition-metal-catalyzed decarboxylation of aromatic carboxylic acids is a known strategy in organic synthesis [134]. This approach can afford unconventional alternatives to perform various types of C–C and C–heteroatom bond-forming reactions. In 2012, Shen and colleagues have achieved an efficient protocol for forming biaryl compounds through palladium-catalyzed intramolecular decarboxylative coupling of arene carboxylic acids with aryl bromides, yielding various cyclized heterocycles, including phenanthridinones [135]. Later, intramolecular decarboxylative amidation of unactivated arenes was achieved under transition metal free conditions (Scheme 20) [136]. Na_2_S_2_O_8_-promoted decarboxylative cyclization of biaryl-2-oxamic acids **37**, gave phenanthridinones **38** with high efficiency. It was observed that the wide scope of the desired products was obtained in good to excellent yields. As electron-withdrawing groups on the aromatic ring did not lower the reaction efficiency, a radical reaction pathway is followed.

## 6. Photo-Mediated Reactions for Phenanthridinone Synthesis

**Scheme 21:**
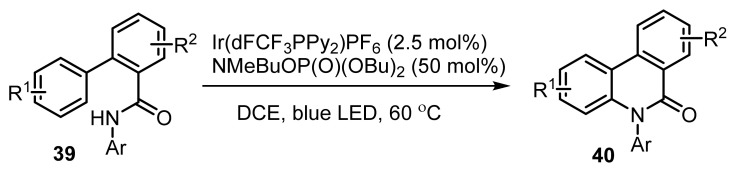
Visible light-induced synthesis of phenanthridinones through direct oxidative C–H amidation.

Recently, photocatalyzed reactions proved to be a valuable method for the synthesis of heterocycles and biologically active compounds [137]. Firstly, Mondon et al. investigated the photocyclization of benzanilides to furnish unsymmetrically substituted phenanthridinones [138]. Later, Prabhakar et al. reported an improved phenanthridinone synthesis by using the photocylization of boron complexes, followed by hydrolysis [139]. In 2017, a facile photo reductive protocol was developed to remove benzyl *o*-protective groups from phenathridin-6-ol via blue light irradiation [140]. Furthermore, Moon and colleagues synthesized phenanthridinones **40** through a visible-light-promoted direct oxidative C–H amidation [141]. In this photocatalytic system, oxidative intramolecular C–H amidation of benzamide **39** was achieved through amidyl radicals. Those could be generated by homolysis of the N–H bond of simple amide precursors via single-electron transfer under blue LED irradiation. The scope of the methodology was further extended to different derivatives of benzamides. Interestingly, halides such as fluoro, chloro, bromo, and iodo substituents were tolerated under the reaction conditions (Scheme 21). Subsequently, the applicability of the method was verified with respect to substrates bearing *p*-methoxyphenyl (PMP) groups on the amide nitrogen. This worked well with the optimized system and provided the desired products easily. When *meta*-substituted substrates were employed, the formation of the C–N bond took place exclusively at the more sterically accessible C–H bond of the aryl ring, affording only one regioisomer.

**Scheme 22:**
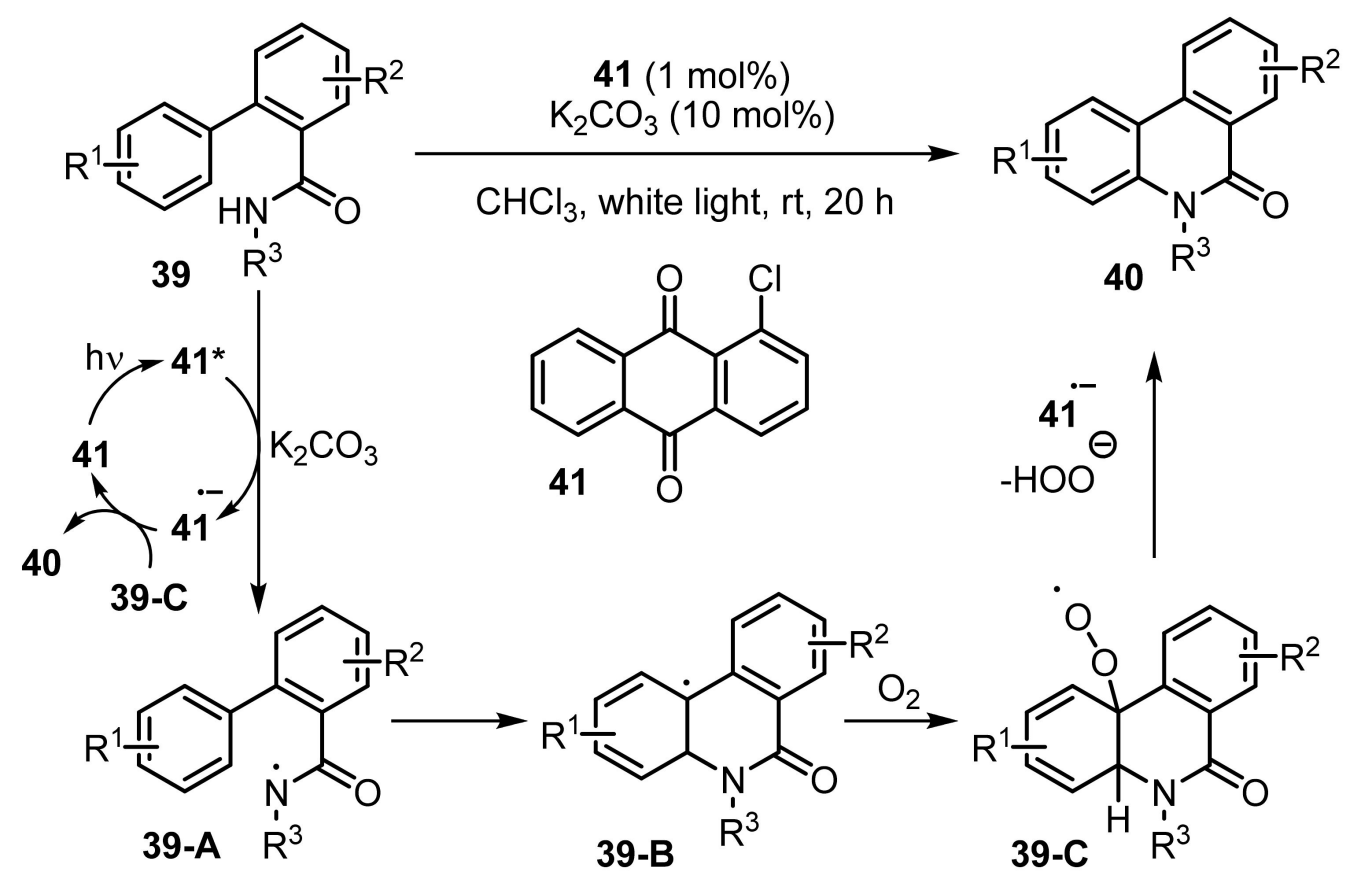
Visible light-induced synthesis of phenanthridinones in the presence of 1- chloroanthraquinone catalyst.

Recently, Itoh et al. developed a transition metal free alternative for visible light-mediated cyclization of benzamides **39** into phenathridinones **40** (Scheme 22) [142]. As a photocatalyst, 1-chloroanthraquinone **41** was employed. The reaction was performed under air atmosphere. The proposed mechanism is similar to the transformation on Scheme 21. Photoexcited catalyst oxidizes benzamide **39** to generate an amidyl radical **39-A**, capable of intramolecular cyclization. The subsequent radical **39-B** traps oxygen, giving peroxy radical **39-C**. The latter is reduced and subsequently aromatized by elimination of hydroperoxide anion. In comparison with the Ir-catalyzed reaction, this transformation tolerates alkyl-substituted amides (R^3^ = Ar, Alk).

## 7. Miscellaneous Reactions

**Scheme 23:**
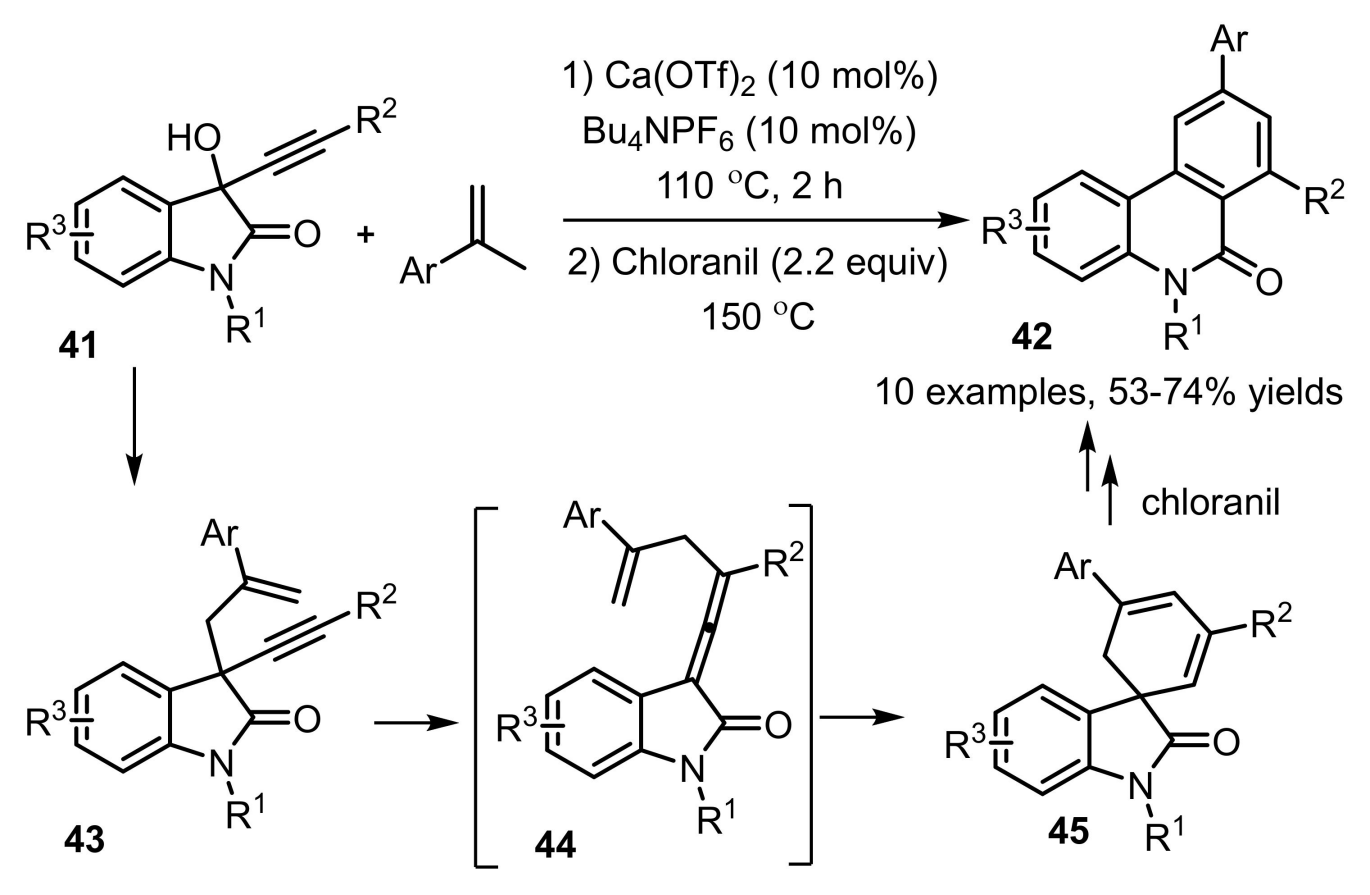
Ca(II)-catalyzed oxidative one-pot domino sequence towards phenathridinones.

In 2017, Yaragorla and colleagues developed a Ca(II)-catalyzed one-pot reaction between oxindole **41** and styrenes, furnishing phenanthridinones **42** in moderate to good yields (Scheme 23) [143]. The reaction starts with a dehydrative cross-coupling, delivering the isolable intermediate **43**. A sigmatropic rearrangement in the latter leads to the formation of allene **44**, capable of intramolecular cyclization into compound **45**. This spiro-cycle **45** could be transformed into the desired phenathridinone **42** under oxidation-induced ring rearrangement.

**Scheme 24:**
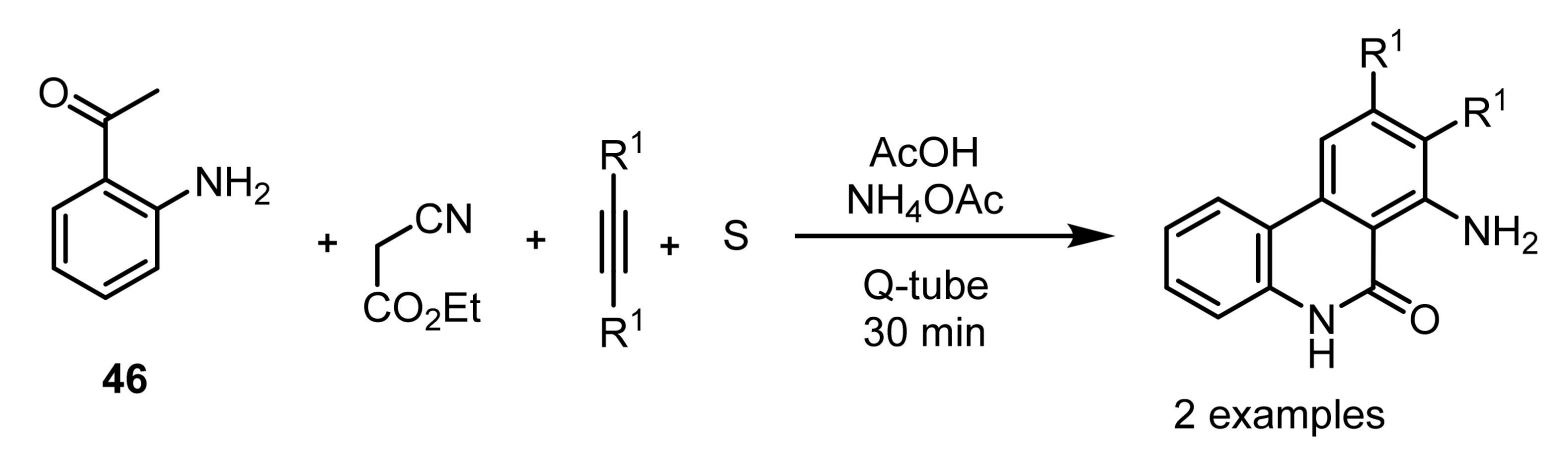
Multicomponent approach towards phenathridinones.

A multicomponent approach for phenathridinone synthesis was developed by Alzaydi and co-workers [144]. The reaction between *ortho*-aminoacetophenone **46**, ethyl cyanoacetate, sulfur and alkyne was carried out in the presence of AcOH/NH_4_OAc under increased pressure in a Q-tube (Scheme 24).

**Scheme 25:**
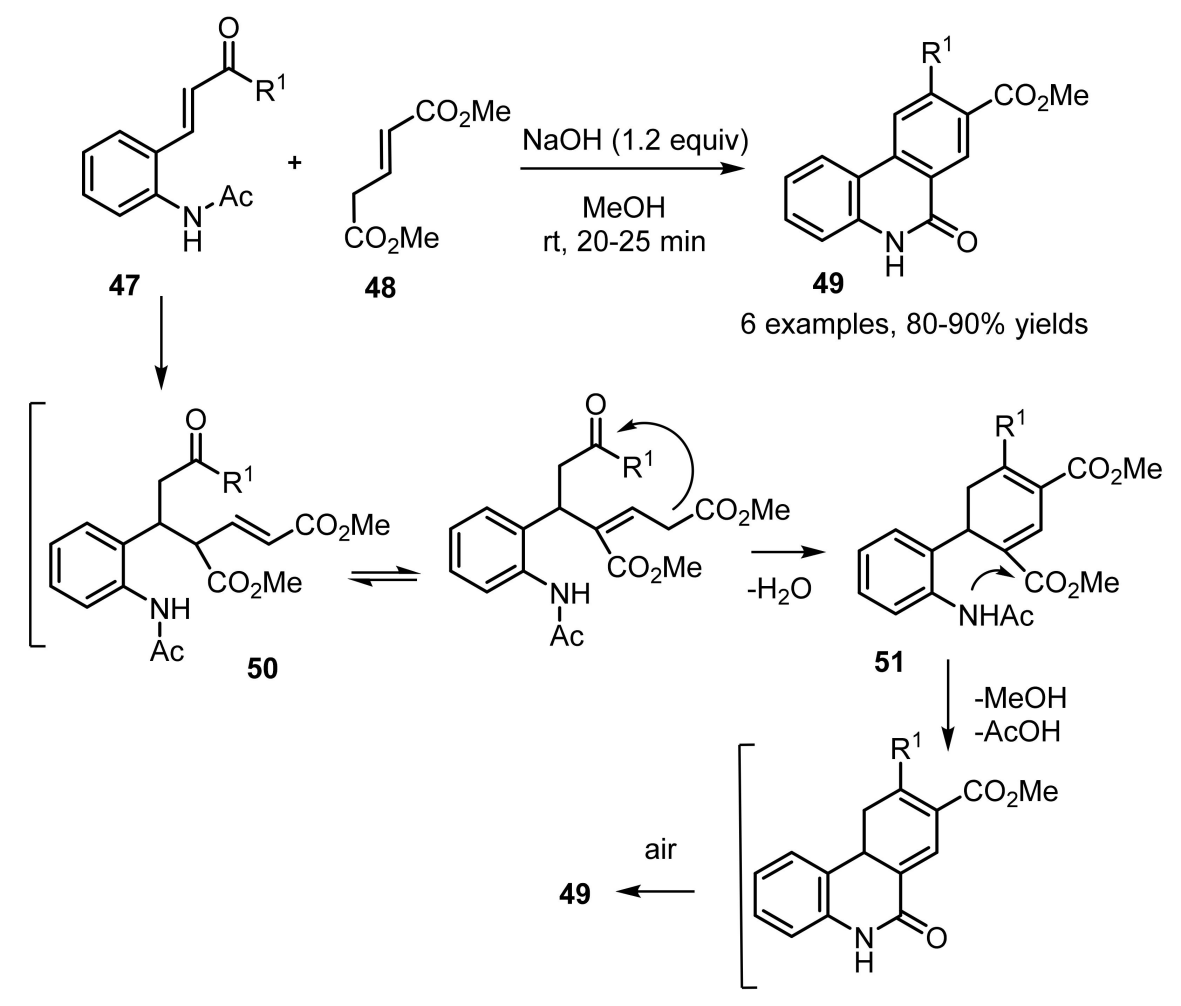
Domino synthesis of phenathridinones from dimethyl glutaconate.

It was shown that unsaturated carbonyl compounds **47** could react with dimethyl glutaconate **48** to produce phenathridinones **49** in excellent yields (Scheme 25) [145]. The reaction took place in methanol at rt under the action of sodium hydroxide. The sequence started with the Michael addition of glutaconate **48** to the activated double bond of **47**, giving intermediate **50**. Double bond migration, followed by intramolecular cyclization gave cyclohexadiene **51**, which underwent an intramolecular cyclization into a non-aromatic phenanthridinone. Spontaneous aromatization delivered the desired product **49**.

## 8. Conclusions

Phenanthridinones have attracted the attention of organic and medicinal chemists because of the diverse pharmacological and biological properties of their alkaloids. Due to promising pharmacological profiles and unique structural features, coupled with low natural abundance, many synthetic methodologies have been developed. Here, we reviewed the synthetic strategies that can achieve phenanthridinone formation. Due to serious efforts in the development of modern synthetic methods, the construction of the phenanthridinone framework through C–C and C–N bond formation reactions via dual C–H bond activation has evolved. Still, the created approaches suffer from the need for pre functionalized substrates, limited substrate scope and scalability, leaving space for new discoveries. We believe that the most crucial developments in sustainable and efficient syntheses of phenathridinones might evolve from the fields of photocatalyzed or electrochemical C–H functionalizations, conceivably combined with the flow chemistry.
